# STAT2/SLC27A3/PINK1-Mediated Mitophagy Remodeling Lipid Metabolism Contributes to Pazopanib Resistance in Clear Cell Renal Cell Carcinoma

**DOI:** 10.34133/research.0539

**Published:** 2024-11-26

**Authors:** Dingheng Lu, Yuxiao Li, Xinyang Niu, Jiazhu Sun, Weitao Zhan, Yuchen Shi, Kai Yu, Suyuelin Huang, Xiaoyan Liu, Liping Xie, Xueyou Ma, Ben Liu

**Affiliations:** ^1^ Department of Urology, The First Affiliated Hospital, Zhejiang University School of Medicine, Hangzhou, 310003 Zhejiang, China.; ^2^ Department of Pathology, The First Affiliated Hospital, Zhejiang University School of Medicine, Hangzhou, 310003 Zhejiang, China.; ^3^ Department of Urology, The First Affiliated Hospital, Zhejiang University School of Medicine, Hangzhou, 310003 Zhejiang, China.; ^4^ Cancer Center, Zhejiang University, Hangzhou, 310003 Zhejiang, China.; ^5^ Department of Urology, The First Affiliated Hospital, Zhejiang University School of Medicine, Hangzhou, 310003 Zhejiang, China.; ^6^ Cancer Center, Zhejiang University, Hangzhou, 310003 Zhejiang, China.

## Abstract

**Background:** Clear cell renal cell carcinoma (ccRCC) is a prevalent malignant tumor of the urinary system. While tyrosine kinase inhibitors (TKIs) are currently the first-line treatments for advanced/metastatic ccRCC, patients often develop resistance after TKI therapy. Lipid metabolic reprogramming, a hallmark of tumor progression, contributes to acquired drug resistance in various malignant tumors. Mitophagy, a process that maintains mitochondrial homeostasis, aids tumor cells in adapting to microenvironmental changes and consequently developing drug resistance. Solute carrier family 27 member 3 (SLC27A3), highly expressed in lipid-rich tumors like ccRCC, has been associated with poor prognosis. However, the impact of SLC27A3 and the transcription factor complex containing STAT2 on lipid metabolic reprogramming, mitophagy in ccRCC, and their role in TKI resistance remain unexplored. **Methods:** 786-O to pazopanib resistance was induced by gradient increase of concentration, and the genes related to lipid metabolism were screened by RNA sequencing. Bioinformatics was used to analyze the differential expression of SLC27A3 and its effect on patient prognosis, and to predict the activated pathway in pazopanib-resistant cells. Lipid droplets (LDs) were detected by Red Oil O and BODIPY probe. Micro-targeted lipidomic of acyl-coenzyme A (CoA) and lipid metabolomics were performed to screen potential metabolites of SLC27A3. The differential expression of SLC27A3 was detected in clinical samples. The differential expression of SLC27A3 and its effect on drug resistance of ccRCC tumor were detected in vitro and in vivo*.* Mitophagy was detected by electron microscopy, Mtphagy probe, and Western blot. The mitochondrial membrane potential (MMP) and reactive oxygen species (ROS) levels were detected by JC-1 and DCF probes. The binding site of the transcription factor complex to the SLC27A3 promoter was detected by dual-luciferase reporter gene assay. **Results:** SLC27A3, highly expressed in lipid-rich tumors such as ccRCC and glioblastoma, predicts poor prognosis. SLC27A3 expression level also increased in pazopanib-resistant 786-O cells (786-O-PR) with more LD accumulation compared to parental cells. Gene Ontology (GO) and Kyoto Encyclopedia of Genes and Genomes (KEGG) pathway analysis from RNA sequencing showed that PINK1/Parkin-mediated mitophagy pathway was enriched in 786-O-PR. Knockdown of SLC27A3 markedly suppressed LD accumulation and mitophagy, and overcame pazopanib resistance *in vitro* and *in vivo*. Moreover, SLC27A3 functions as an acyl-CoA ligase catalyzing the formation of acyl-CoA, which refers to fatty acid oxidation accompanied by ROS production and synthesis of lipid. Overproduced acyl-CoA oxidation in mitochondria resulted in MMP decrease and amounts of ROS production, subsequently triggering PINK1/Parkin-mediated mitophagy. Moreover, mitophagy inhibition led to more ROS accumulation and cell death, indicating that mitophagy can keep ROS at an appropriate level by negative feedback. Mitophagy, simultaneously, prevented fatty acid oxidation in mitochondria by consuming CPT1A, forcing synthesis of triglycerides and cholesterol esters stored in LDs by transforming acyl-CoA, to support ccRCC progression. Besides, we found that STAT2 expression was positively correlated to SLC27A3. Transcriptional factor complex containing STAT2 could bind to the promoter of SLC27A3 mRNA to promote SLC27A3 transcription proved by dual-luciferase reporter assay, which also regulated LD metabolism and activated mitophagy during pazopanib resistance. **Conclusion:** SLC27A3 is up-regulated in pazopanib-resistant ccRCC and predicts poor prognosis. High expression of SLC27A3 produces excessive metabolites of various long-chain fatty acyl-CoA (12:0-, 16:0-, 17:0-, 20:3-CoA) to enter mitochondria for β-oxidation and produce amounts of ROS activating mitophagy. Subsequent mitophagy/ROS negative feedback controls ROS homeostasis and consumes CPT1A protein within mitochondria to suppress fatty acid β-oxidation, forcing acyl-CoA storage in LDs, mediating pazopanib resistance in ccRCC. Furthermore, STAT2 was identified as a core component of a potential upstream transcriptional factor complex for SLC27A3. Our findings shed new light on the underlying mechanism of SLC27A3 in ccRCC TKI resistance, which may provide a novel therapeutic target for the management of ccRCC.

## Introduction

Renal cell carcinoma (RCC) is a malignant tumor of the urinary system, with clear cell renal cell carcinoma (ccRCC) being the most common type, accounting for approximately 75% of renal malignant tumors [1,2]. Surgical treatment can yield satisfactory results in localized tumors. Given that ccRCC is not sensitive to radiotherapy or chemotherapy, immune checkpoint inhibitors (ICIs; such as pembrolizumab) and tyrosine kinase inhibitors (TKIs; such as sunitinib and pazopanib) remain the mainstay of treatment for advanced/metastatic ccRCC [3]. However, advanced ccRCC often develops resistance to TKI therapy within 6 to 12 months [4]. Understanding the mechanisms underlying ccRCC TKI resistance is crucial for developing more effective treatments.

Lipid metabolic reprogramming plays a critical role in tumor progression [5,6]. Characteristically, ccRCC is characterized by the accumulation of lipid droplets (LDs), partially resulting from disruptions in the von Hippel Lindau–hypoxia-inducible factor (VHL-HIF) pathway [7,8]. Recent studies have revealed that LDs in ccRCC are primarily synthesized from free fatty acids (FFAs), glycerol, and sterols [9–11]. VHL dysfunction-induced HIF accumulation can inhibit the expression of carnitine palmitoyltransferase 1A (CPT1A) on the mitochondrial membrane, preventing FFA oxidation and promoting the storage of FFAs in LDs. This subsequently provides raw materials for cell proliferation in ccRCC [12,13]. Moreover, lipid metabolic reprogramming contributes to acquired drug resistance in various malignant tumors [14]. Increased FA uptake and enhanced β-oxidation have been implicated in mediating cisplatin resistance in lung cancer [15], while increased FA oxidation can promote acquired resistance to BRAF inhibitors in melanoma [16].

Mitophagy, a selective autophagy process, maintains metabolic homeostasis by eliminating dysfunctional mitochondria. The PINK1/Parkin pathway has been extensively studied in this regard. It is now understood that when the mitochondrial membrane potential (MMP) is damaged, PINK1 anchors to the outer membrane to recruit Parkin, leading to mitophagy [17]. Mitochondrial homeostasis aids tumor cells in adapting to the changing microenvironment and developing drug resistance [18,19]. PRCC-TFE3 translocation RCC is known to activate mitophagy by up-regulating Parkin expression, clearing oxidation-damaged mitochondria for cell survival [20]. PINK1-mediated mitophagy can promote acquired drug resistance in hepatocellular carcinoma cells [21] or induce drug-tolerant persister formation in lung adenocarcinoma [22]. As the primary site of fatty acid oxidation, the mitochondrion is closely linked to lipid metabolic reprogramming. High diacylglycerolacyltransferase (DGAT) expression in glioblastoma reportedly promotes the storage of excess FFAs in LDs to maintain lipid homeostasis. DGAT inhibition leads to FFA oxidation accompanied by increased reactive oxygen species (ROS), inducing glioblastoma cell apoptosis [23]. Therefore, mitophagy is anticipated to remove excess ROS and regulate β-oxidation rates during drug resistance development.

The solute carrier family 27 member 3 (*SLC27A3*) gene is situated on chromosome 1q21.3, and its encoded protein primarily functions as an acyl-coenzyme A (CoA) ligase, catalyzing the biosynthesis of acyl-CoA from long-chain fatty acids (LCFAs) [24]. Fatty acids must be activated into acyl-CoA to participate in various lipid metabolic processes, including β-oxidation, lipid synthesis, fatty acid modification, posttranslational modification, and transcriptional regulation. Acyl-CoA synthetase catalyzes this activation reaction [25,26]. Mutations in SLC27A3 have been linked to autism spectrum disorders [27], and its wild-type form has been shown to activate both C24:0 and C16:0 fatty acids in mouse models [28]. Additionally, SLC27A3 plays a crucial role in tumorigenesis and cancer progression, as exemplified in glioma [24], lung cancer [29], and glioblastoma [30]. Nonetheless, the impact of *SLC27A3* on lipid metabolic reprogramming and mitophagy in ccRCC, along with its potential role in TKI resistance, has not been previously reported.

There are currently limited studies on SLC27A3, particularly regarding its regulatory mechanisms. Signal transducer and activator of transcription 2 (STAT2), a member of the STAT transcription factor family, is implicated in regulating tumor development [31,32]. STAT2 promotes chemotherapy resistance in ovarian cancer [31] and remodels lipid metabolism during colorectal cancer progression [32]. In conjunction with STAT1, STAT2 forms a transcription factor complex to mediate the transcription of downstream genes [33]. In this study, we observed a positive correlation between STAT2 and SLC27A3 expression in ccRCC and established a stable pazopanib-resistant 786-O cell line (786-O-PR) with up-regulated SLC27A3. High SLC27A3 expression was associated with a poorer prognosis. Specifically, increased SLC27A3 expression enhanced mitochondrial fatty acid β-oxidation accompanied by ROS production, activating PINK1/Parkin-mediated mitophagy. Subsequent negative feedback from mitophagy could regulate ROS levels and prevent further mitochondrial β-oxidation progression by restricting CPT1A, forcing acyl-CoA storage in LDs, ultimately promoting pazopanib resistance in ccRCC.

## Results

### SLC27A3 up-regulation in the newly established ccRCC cell lines and its association with prognosis

**Fig. 1.**
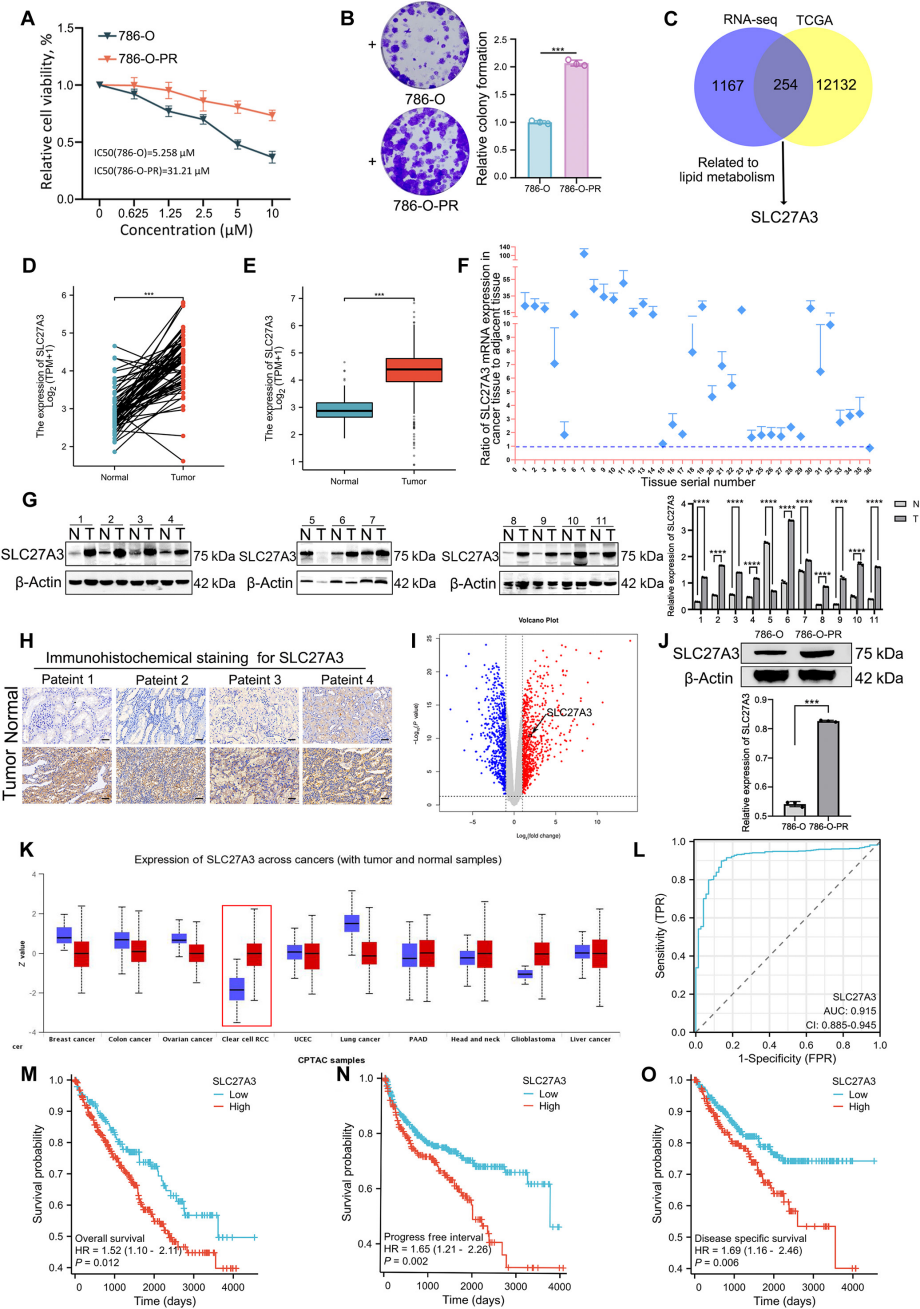
SLC27A3 up-regulation in the newly established ccRCC cell lines and its association with prognosis. (A) IC_50_ values of 786-O and 786-O-PR cells. (B) Proliferation ability of 786-O and 786-O-PR cells was detected by colony formation assay. (C) Overlap TCGA-KIRC up-regulated gene data and RNA-sequencing results from 786-O and 786-O-PR and select SLC27A3. (D and E) SLC27A3 was highly expressed in tumor tissues according to the TCGA-KIRC database. (F) SLC27A3 mRNA levels in 36 ccRCC tissues and adjacent normal tissues (the control group: the mRNA level of normal tissues). (G) Western blot experiments for SLC27A3 protein level were performed in tumor and adjacent tissues collected from 11 patients with ccRCC (N, normal; T, tumor). (H) The expression of SLC27A3 was identified by IHC in normal and tumor tissues from collected samples in the hospital. (I) RNA-sequencing results showed that SLC27A3 was up-regulated in 786-O-PR cells compared with parental cells. (J) Western blot experiment results showed that SLC27A3 was up-regulated in 786-O-PR cells compared with parental ones. (K) Pan-cancer analysis revealed that SLC27A3 was significantly overexpressed in lipid-rich tumors. (L) ROC curve analysis showed that SLC27A3 expression is specific in ccRCC. (M to O) OS, PFI, and DSS were analyzed according to high or low expression of SLC27A3, and the log-rank *t* test was used (*P < 0.05, **P < 0.01, ***P < 0.001).

Figure 1A displays the IC_50_ values of both the parental 786-O and 786-O-PR cell lines. Cell viability assays revealed that 786-O-PR cells proliferated more slowly than did 786-O cells (Fig. S1A) and exhibited cell cycle arrest in the G_2_ stage (Fig. S1B). Upon pazopanib treatment, 786-O cells proliferated more slowly than did 786-O-PR cells (Fig. 1B and Fig. S1C). 786-O cells were arrested in the G_1_ stage, suggesting cell viability suppression, while no obvious effect of pazopanib on 786-O-PR was observed (Fig. S1D). By overlapping TCGA (The Cancer Genome Atlas)–KIRC (Kidney Clear Cell Carcinoma) up-regulated genes and RNA sequencing data from 786-O and 786-O-PR, SLC27A3 was selected based on its marked differential expression related to lipid metabolism and its crucial role in ccRCC (Fig. 1C). Both paired (Fig. 1D) and unpaired (Fig. 1E) analyses of KIRC-TCGA revealed elevated SLC27A3 expression in tumor samples. Similar results were obtained in the independent dataset (Fig. 1F) and Gene Expression Omnibus (GEO) analysis of GDS505 (Fig. S1E) [34]. Consistently, SLC27A3 protein levels were increased in clinical tumor samples compared to adjacent normal tissues from 11 ccRCC patients (Fig. 1G) and in cell lines (Fig. S1F). Furthermore, higher SLC27A3 expression was detected in clinical specimens using immunohistochemistry (IHC) (Fig. 1H and Fig. S1G) and the Human Protein Atlas (THPA) database (Fig. S1H). Additionally, we found that SLC27A3 was up-regulated in 786-O-PR cells compared to parental cells in RNA sequencing data (Fig. 1I) and protein levels (Fig. 1J), suggesting a potential role of SLC27A3 in TKI resistance progression.

Pan-cancer analysis revealed that *SLC27A3* was overexpressed in lipid-rich tumors such as ccRCC and glioblastoma (Fig. 1K). SLC27A3 was also found to be highly important in the diagnosis of ccRCC with area under curve (AUC) = 0.915 (Fig. 1L), indicating its specificity in ccRCC. Moreover, KIRC-TCGA analysis demonstrated that patients with higher SLC27A3 expression exhibited worse overall survival (OS), progression-free interval (PFI), and disease-specific survival (DSS) compared to those with lower *SLC27A3* expression (Fig. 1M to O). Collectively, these findings suggest that SLC27A3 is up-regulated in ccRCC and TKI-resistant cells, is associated with lipid metabolism, and indicates a poorer prognosis.

### SLC27A3 mediates TKI resistance in ccRCC by regulating LDs

**Fig. 2.**
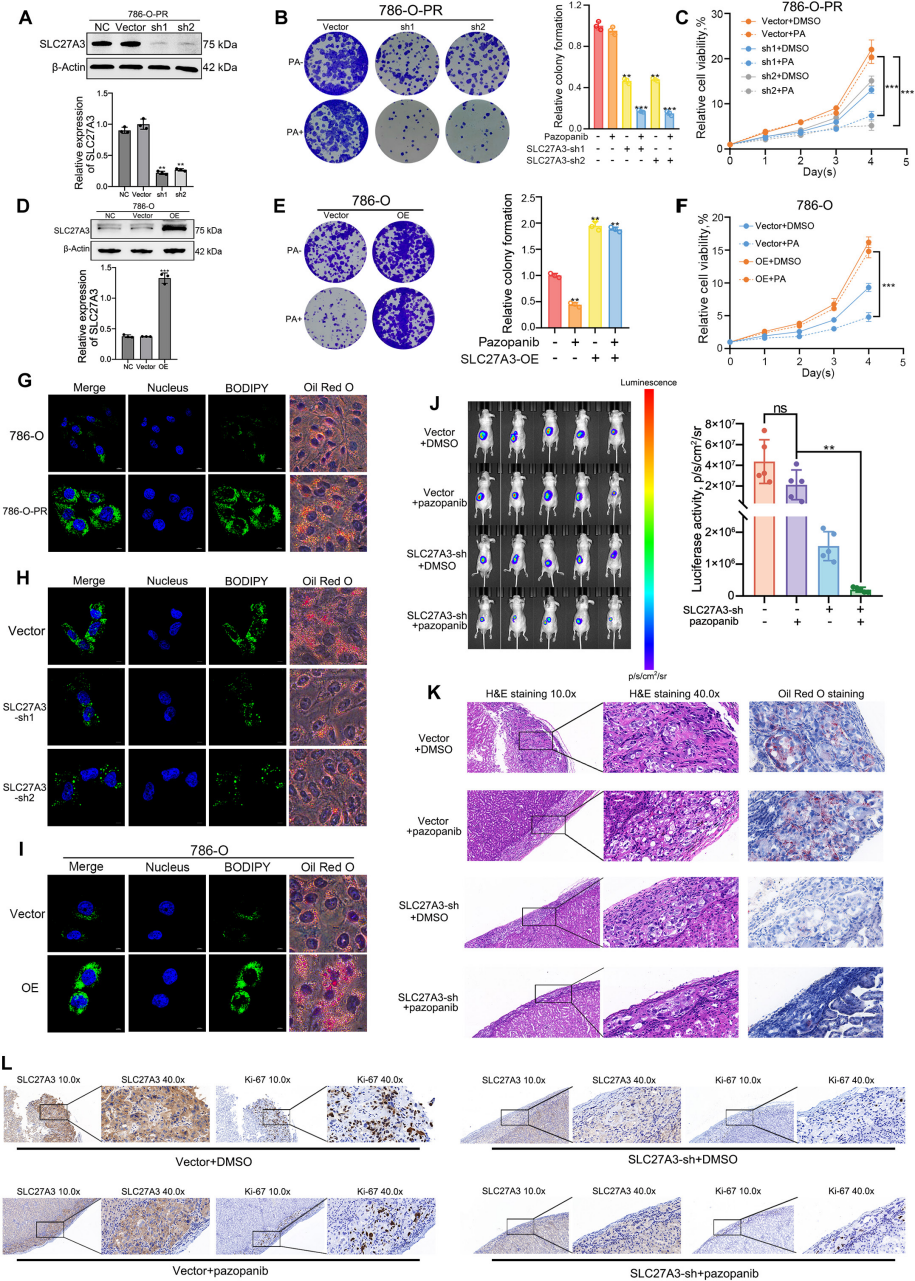
SLC27A3 mediates TKI resistance in ccRCC by regulating LDs. (A to C) Protein expression levels, relative colony formation, and relative cell viabilities detected by Western blot, colony formation assay, and CCK-8 assay in 786-O-PR cells after transfection with sh1-SLC27A3 or sh2-SLC27A3 plasmids. (D to F) Protein expression levels, relative colony formation, and relative cell viabilities detected by Western blot, colony formation assay, and CCK-8 assay in 786-O cells after transfection with SLC27A3-OE plasmids. (G) LD in 786-O and 786-O-PR cells detected by BODIPY staining and Oil Red O staining. (H) LD in the SLC27A3-sh1 and SLC27A3-sh2 transfection groups and the control group detected by BODIPY and Oil Red O staining. (I) LD of 786-O in the SLC27A3-OE transfection groups and the control group detected by BODIPY and Oil Red O staining. (J) Representative image and fluorescence intensity of renal orthotopic tumors in nude mice (*n* = 5 per group). (K) Hematoxylin and eosin (H&E) staining, along with Oil Red O staining, was utilized to assess both the quantity and dimensions of tumor formations in vivo. (L) IHC staining of SLC27A3 and Ki-67 was utilized to assess tumor growth in vivo*.*

As shown in Fig. 2A and Fig. S2H, after transfection with SLC27A3-sh1 and SLC27A3-sh2 plasmids, 786-O-PR cells exhibited reduced SLC27A3 expression levels with no difference between negative control (NC) and Vector. Colony formation and CCK-8 assays demonstrated that SLC27A3 knockdown groups displayed decreased colony formation ability and relative cell viability in the presence of pazopanib (Fig. 2B and C). To further investigate, we overexpressed SLC27A3 in parental cells (Fig. 2D and Fig. S2A). Both colony formation ability and relative cell viability were enhanced after SLC27A3 overexpression in the presence of pazopanib (Fig. 2E and F and Fig. S2B and C). Given SLC27A3’s association with lipid metabolism, BODIPY probe and Oil Red O staining were performed. Pazopanib-resistant cells were observed to have an abundance of LDs compared to parental cells (Fig. 2G). Subsequently, SLC27A3 knockdown in 786-O-PR cells resulted in a reduction of LDs (Fig. 2H). Conversely, SLC27A3 overexpression in parental cells led to an increase in LD formation (Fig. 2I and Fig. S2D). Furthermore, considering that the primary components of LDs are triglycerides (TGs) and cholesterol esters (CEs), the addition of DGAT1/2i and SOAT1/2i [LD inhibitor cocktail (LDIC)] markedly inhibited LD formation and pazopanib resistance (Fig. S2E to G).

To investigate the in vivo impact of SLC27A3 on TKI resistance in ccRCC*,* 786-O-PR cells infected with SLC27A3 knockdown lentivirus were selected and injected into distinct groups *in situ*. In the presence of pazopanib, an obvious decrease in tumor luciferase activity was observed in the SLC27A3 knockdown group (Fig. 2J and Fig. S2I), accompanied by a reduction in tumor size (Fig. 2K and Fig. S2I). IHC data demonstrated that SLC27A3 knockdown resulted in weaker tumor proliferation ability in the presence of pazopanib (Fig. 2L and Fig. S2J). In conclusion, the findings presented above confirmed that SLC27A3 can mediate TKI resistance in ccRCC by regulating LD synthesis.

### Mitophagy can mediate TKI resistance in ccRCC by regulating LDs

**Fig. 3.**
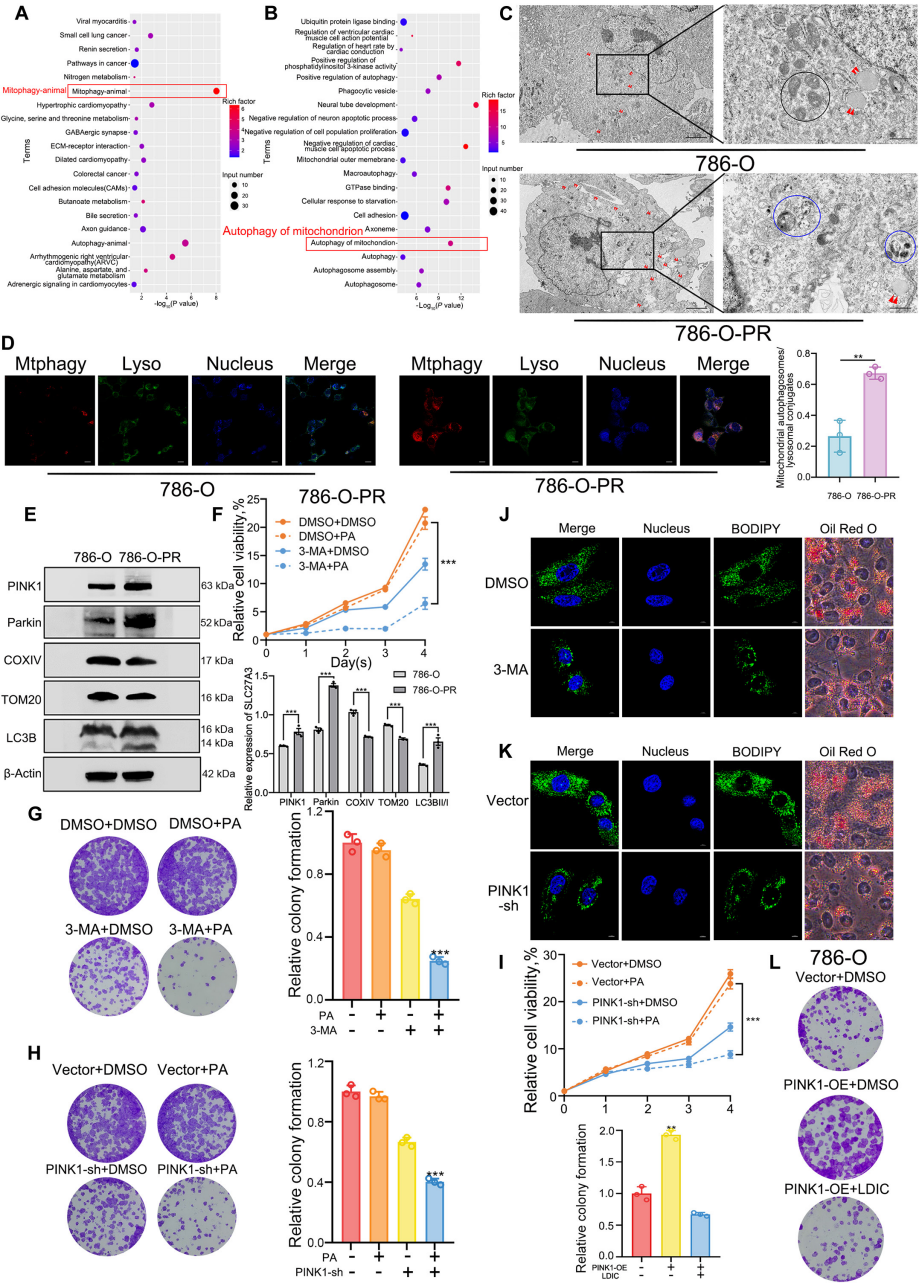
Mitophagy can mediate TKI resistance in ccRCC by regulating LDs. (A and B) KEGG and GO enrichment analysis between 786-O-PR versus 786-O cells. (C) TEM manifested that 786-O-PR cells showed more LDs and mitophagy phenomenon than 786-O cells (original magnification, ×1,200 and ×5,000, respectively). Red arrow, LD; blue circle, mitophagy; black circle, normal mitochondria. (D) More mitophagy phenomena and lysosome activation were detected by Mtphagy and Lyso probes in 786-O-PR cells. (E) Expression levels of mitophagy-related proteins between 786-O and 786-O-PR cells. (F) Relative cell viabilities of 786-O-PR treated with 3-MA were detected by CCK-8. (G and H) Proliferation ability of 786-O-PR treated with 3-MA or stably transfected with PINK1-sh plasmids or vectors was detected by colony formation assay. (I) Relative cell viabilities of 786-O-PR stably transfected with PINK1-sh plasmids were detected by CCK-8. (J and K) LD in the 3-MA treatment group or the stable PINK1-sh transfection group as well as the control groups detected by BODIPY staining and Oil Red O staining. (L) Proliferation ability of 786-O stably transfected with PINK1-OE plasmids or vectors or treated with LDIC was detected by colony formation assay.

Both Kyoto Encyclopedia of Genes and Genomes (KEGG) pathway and Gene Ontology (GO) analyses for 786-O-PR and 786-O samples revealed enrichment for the mitophagy pathway (Fig. 3A and B). Under transmission electron microscopy (TEM), 786-O-PR cells exhibited more LDs and autophagosomes containing mitochondria (Fig. 3C). Mitophagy phenomena and lysosome activation were more prominent in 786-O-PR cells after treatment with Mtphagy Dye (Fig. 3D). Western blot analysis yielded similar results. Key mitochondrial proteins, COXIV and TOM20, were down-regulated in 786-O-PR cells, indicating a reduction in the number of mitochondria. The ratio of LC3B-II to LC3B-I was increased in 786-O-PR cells, along with PINK1 and Parkin protein levels (Fig. 3E). These findings collectively suggest that mitophagy was activated in 786-O-PR cells.

Although pazopanib alone had a limited suppressive effect on the proliferation ability of 786-O-PR cells, this effect was inhibited by pazopanib in combination with the mitophagy inhibitor 3-methyladenine (3-MA) (Fig. 3F). Additionally, colony formation results demonstrated that adding 3-MA impaired the proliferation ability of 786-O-PR cells under pazopanib conditions (Fig. 3G). Similarly, PINK1 knockdown inhibited the proliferation of 786-O-PR cells in the presence of pazopanib (Fig. 3H and I). As shown in Fig. 3J, under BODIPY probe and Oil Red O staining, cells treated with 3-MA exhibited fewer LDs, and cells stably transfected with PINK1-sh plasmids behaved similarly (Fig. 3K). To further confirm the effect of mitophagy on TKI resistance in ccRCC, we overexpressed PINK1 in parental cells. Colony formation results validated that cells with PINK1 overexpression exhibited a higher survival rate, while those treated with LDIC displayed a lower colony formation rate in the presence of pazopanib, indicating the crucial role of LDs in TKI resistance (Fig. 3L and Fig. S3A). The effects of PINK1-sh and PINK1-OE on mitophagy were validated in Fig. S3B to D.

### SLC27A3 mediates mitophagy by regulating ROS levels, affecting LD formation and TKI resistance in ccRCC

**Fig. 4.**
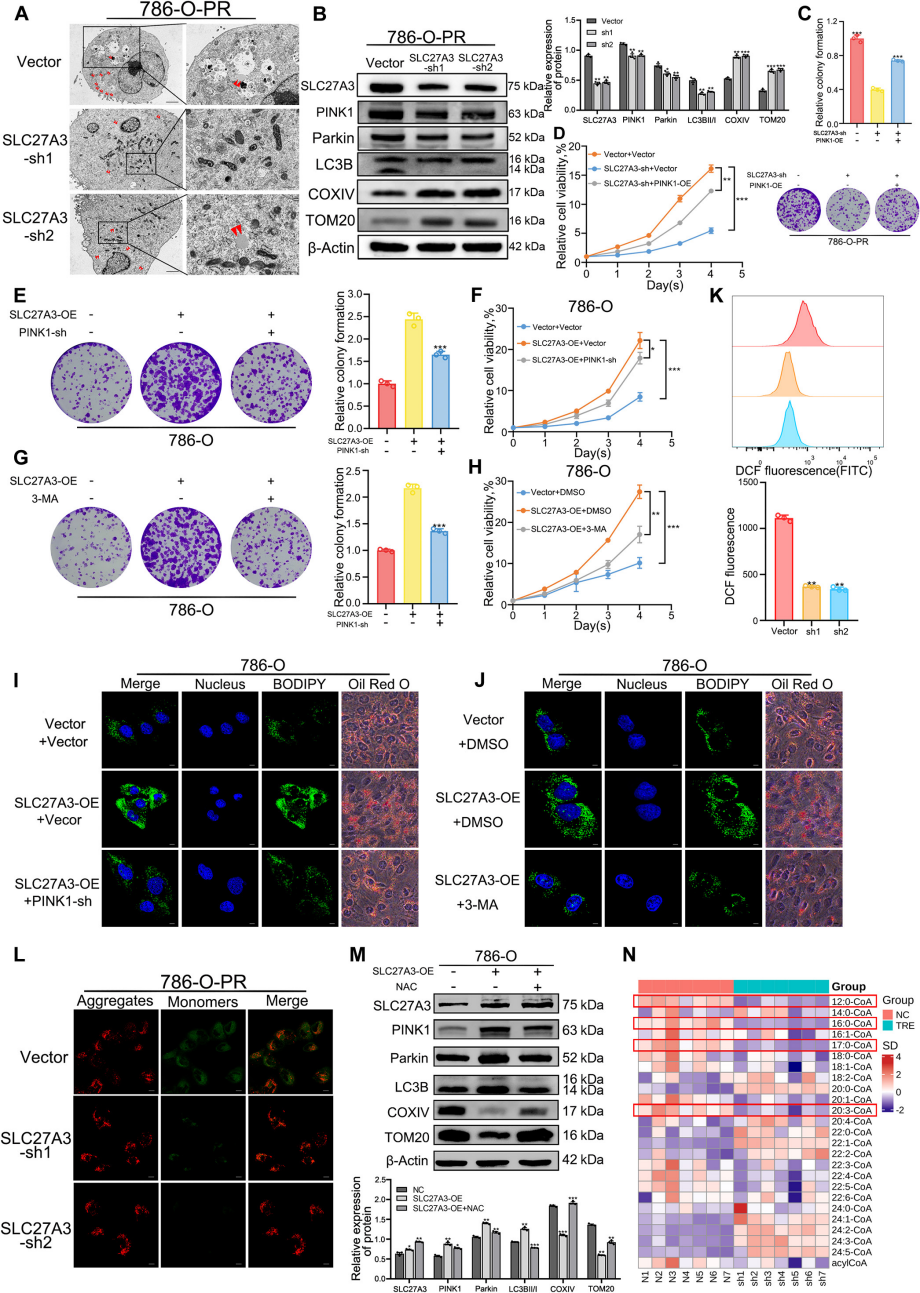
SLC27A3 mediates mitophagy by regulating ROS levels, affecting LD formation and TKI resistance in ccRCC. (A) TEM analysis revealed a reduction in mitophagy in 786-O-PR cells following SLC27A3 knockdown (original magnification, ×1,200 and ×5,000, respectively). Red arrow, LD. (B) Expression levels of mitophagy-related proteins between 786-O-PR and 786-O-PR cells with SLC27A3 knocked down. (C and D) Proliferation ability of 786-O-PR cells stably transfected with PINK1-OE plasmids, or SLC27A3-sh was detected by colony formation and CCK-8 assays. (E and F) Proliferation ability of 786-O cells stably transfected with SLC27A3-OE plasmids or PINK1-sh was detected by colony formation and CCK-8 assays. (G and H) Proliferation ability of 786-O cells stably transfected with SLC27A3-OE plasmids or added with 3-MA was detected by colony formation and CCK-8 assays. (I) LD in the SLC27A3-OE treatment group, SLC27A3-OE cotreatment group with PINK1-sh, and the control group detected by BODIPY and Oil Red O staining. (J) LDs in the SLC27A3-OE treatment group, SLC27A3-OE cotreatment group with 3-MA, and the control group detected by BODIPY and Oil Red O staining. (K) The DCF fluorescence level of ROS was detected by flow cytometry. (L) MMP levels were measured by JC-1 fluorescence probe. (M) Expression level of mitophagy-related proteins in 786-O cells tested by Western blot. (N) Lipomic analysis of long-chain fatty acyl-CoA between SLC27A3 knockdown and control group in pazopanib-resistant cells.

To further investigate the role of SLC27A3 in mitophagy and TKI resistance, we down-regulated SLC27A3 in 786-O-PR cells using SLC27A3-specific short hairpin RNA (shRNA). TEM revealed reduced mitophagy activity (Fig. 4A). Western blot analysis demonstrated that SLC27A3 knockdown clearly decreased PINK1 and Parkin expression levels, as well as the LC3B-II to LC3B-I ratio. Mitochondrial proteins COXIV and TOM20 remained unchanged, indicating the preservation of mitochondrial volume (Fig. 4B). Additionally, Mtphagy Dye staining revealed fewer mitophagy events in SLC27A3-sh groups (Fig. S4A). Proliferation assays showed that SLC27A3 knockdown resulted in lower survival rates in 786-O-PR cells in the presence of pazopanib, while colony formation ability could be rescued by subsequent overexpression of PINK1 (Fig. 4C) and cell viability (Fig. 4D). To validate this, we conducted a similar experiment in parental cells. We observed that overexpressing SLC27A3 in parental cells augmented colony formation ability (Fig. 4E and Fig. S4B) and cell viability (Fig. 4F and Fig. S4C). However, subsequent down-regulation of PINK1 could rescue this effect. Similarly, replacing PINK1-sh with 3-MA yielded similar outcomes (Fig. 4G and H and Fig. S4D and E). Regarding the correlation between SLC27A3 and LD formation, we induced SLC27A3 overexpression in 786-O and 769-P cells, coupled with either PINK1 knockdown (Fig. 4I and Fig. S4F) or the addition of 3-MA (Fig. 4J and Fig. S4G), which inhibited LD formation.

ROS are considered intermediaries between SLC27A3 and mitophagy, serving as by-products of β-oxidation and activators of mitophagy [35]. Compared to the control group, ROS levels were markedly reduced in SLC27A3 knockdown groups (Fig. 4K). Additionally, we assessed MMP using the specific JC-1 probe. The results revealed a higher red-to-green fluorescence ratio and increased MMP in SLC27A3 knockdown groups, indicating a decrease in ROS (Fig. 4L). We also overexpressed SLC27A3 in parental cells. Western blot analysis showed that SLC27A3 overexpression mediated mitophagy activation, which could be rescued by ROS clearance with N-acetylcysteine (NAC) (Fig. 4M and Fig. S4J). Moreover, in the presence of pazopanib, the 786-O and 769-P cell lines with SLC27A3 up-regulation exhibited higher survival rates, which were inhibited by the addition of NAC (Fig. S4H and I). Furthermore, micro-targeted lipidomic analysis of acyl-CoAs was conducted (Fig. S4K). C12:0-, C16:0-, C17:0-, and C20:3-CoA were found to be decreased in the SLC27A3 knockdown group, suggesting that these LCFAs might be substrates of SLC27A3 (Fig. 4N and Fig. S4L to O). Nontargeted lipid metabolomics identified diacylglycerol [16:0/18:1(11Z)/0:0] as one of the core metabolites (Fig. S4P).

### Mitophagy exerts a negative regulatory influence on CPT1A and ROS levels, consequently modulating the process of lipid biosynthesis

**Fig. 5.**
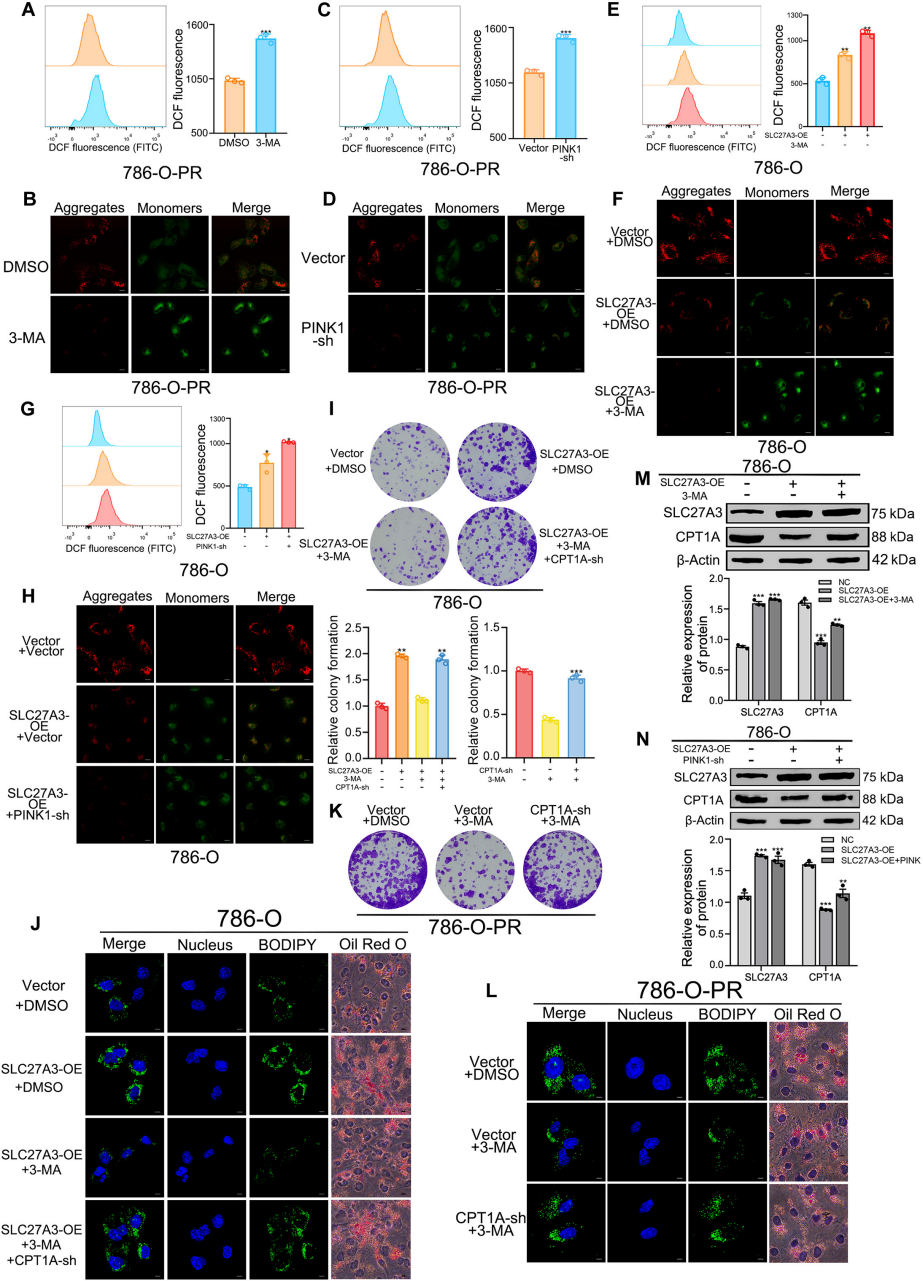
Mitophagy exerts a negative regulatory influence on CPT1A and ROS levels, consequently modulating the process of lipid biosynthesis. (A to D) ROS and MMP levels detected by flow cytometry and JC-1 fluorescence probe of the 786-O-PR cells added with 3-MA or PINK1-sh and the control groups. (E to H) ROS and MMP levels detected by flow cytometry and JC-1 fluorescence probe of the 786-O cells transfected with SLC27A3-OE and added with 3-MA or PINK1-sh and the control groups. (I) Effects of SLC27A3-OE, 3-MA, and CPT1A-sh on relative colony formation of 786-O cells. (J) Effects of SLC27A3-OE, 3-MA, and CPT1A-sh on LD synthesis ability of 786-O cells detected by BODIPY and Oil Red O staining. (K) Effects of 3-MA and CPT1A-sh on relative colony formation of 786-O-PR cells. (L) Effects of 3-MA and CPT1A-sh on LD synthesis ability of 786-O-PR cells detected by BODIPY and Oil Red O staining. (M and N) Expression levels of SLC27A3 and CPT1A detected by Western blot experiments in 786-O transfected with SLC27A3-OE and added with 3-MA or PINK1-sh.

We next transfected 786-O-PR cells with the PINK1-sh plasmid or added 3-MA, resulting in a notable increase in ROS levels and a concomitant decrease in MMP levels (Fig. 5A to D). Similar experiments were conducted in parental cells. SLC27A3 overexpression led to elevated ROS levels and decreased MMP levels. Subsequent treatment with either 3-MA or PINK1-sh further intensified ROS accumulation and exacerbated MMP reduction (Fig. 5E to H and Fig. S5A to D). To explore the potential role of CPT1A, we designed the following experiments. In the presence of pazopanib, SLC27A3 overexpression combined with 3-MA addition in parental cells impaired proliferation ability and LD synthesis ability, which could be restored by further transfection with CPT1A-sh (Fig. 5I and J and Fig. S5E and F). Similarly, the addition of 3-MA to 786-O-PR reduced colony formation ability and LD synthesis. Subsequent transfection with CPT1A-sh recovered and improved these parameters (Fig. 5K and L). Western blot assays revealed that SLC27A3 overexpression in 786-O and 769-P cells resulted in a reduction of CPT1A protein levels. Subsequently, the addition of either 3-MA or PINK1-sh rescued CPT1A protein levels through negative feedback regulation (Fig. 5M and N and Fig. S5G and H).

### The transcription factor STAT2 influences the expression of SLC27A3 and downstream biological behaviors

**Fig. 6.**
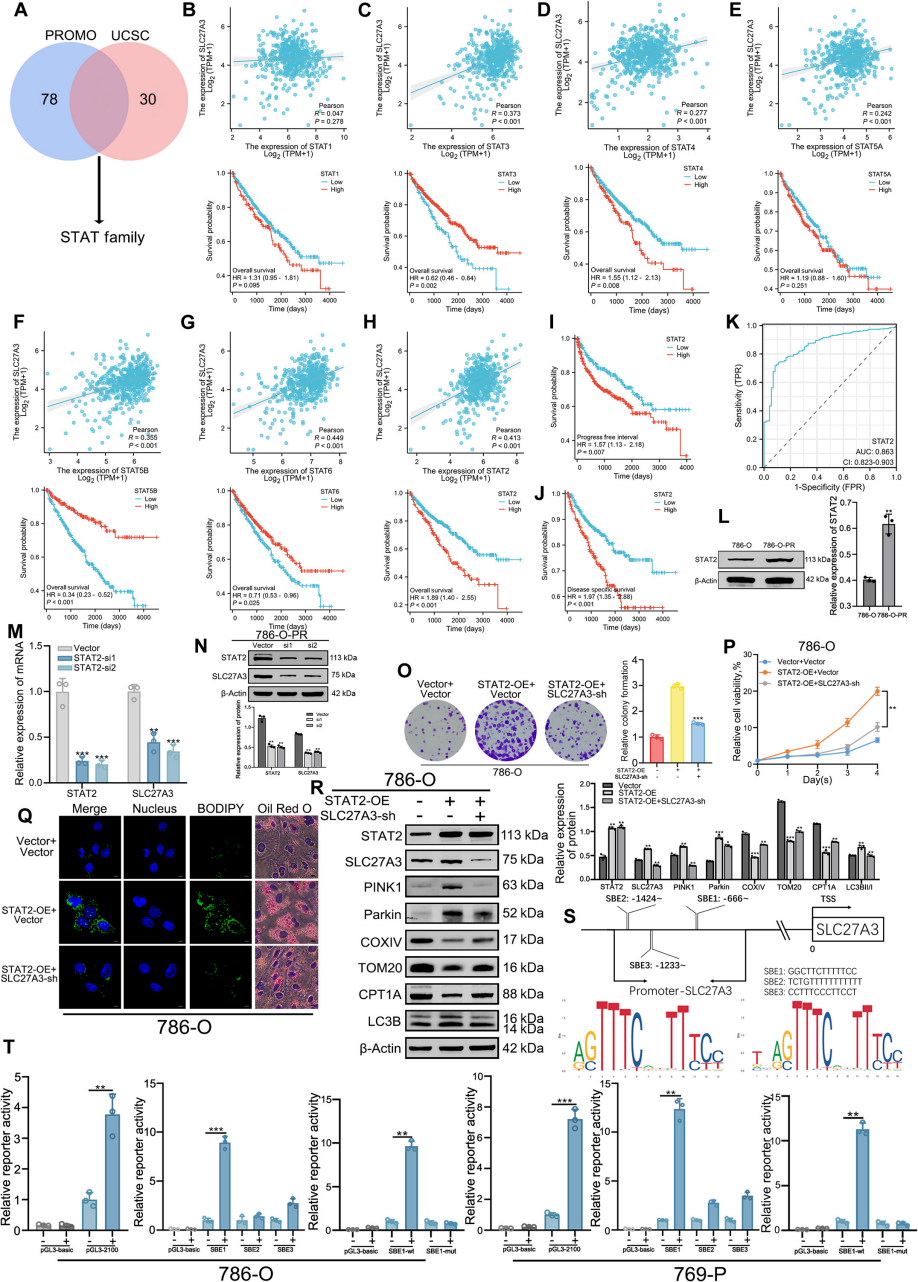
The transcription factor STAT2 influences the expression of SLC27A3 and downstream biological behaviors. (A) STAT family was selected from the PROMO and UCSC databases. (B to H) Pearson correlation analysis of STAT family and SLC27A3 expression levels and clinical OS curves of STAT family members. (I to K) PFI analysis, DSS analysis, and ROC curve of STAT2 in KIRC-TCGA database. (L) Protein expression levels of STAT2 in both 786-O and 786-O-PR cells detected by Western blot experiment. (M) The mRNA expression level of SLC27A3 decreased after knocking down STAT2. (N) The protein expression level of SLC27A3 decreased after knocking down STAT2. (O and P) Proliferation ability of 786-O cells stably transfected with STAT2-OE plasmids and SLC27A3-sh was detected by colony formation and CCK-8 assays. (Q) LD in the STAT2-OE treatment group, STAT2-OE cotreatment group with SLC27A3-sh, and the control group of 786-O cells detected by BODIPY staining and Oil Red O staining. (R) Expression levels of mitophagy-related proteins in 786-O cells detected by Western blot. (S) Transcription factor binding sites were confirmed and analyzed in the JASPAR database, screening the most suitable transcriptional factor binding site. (T) Dual-luciferase reporter assays on SBE1, SBE2, and SBE3 binding site of the SLC27A3 promoter in 786-O and 769-P cell lines.

To elucidate the mechanism of SLC27A3 up-regulation, we screened the STAT family using both the PROMO and UCSC databases (Fig. 6A). Subsequently, we individually investigated the correlation between SLC27A3 and STAT family members’ expression, including STAT1 (Pearson *R* = 0.047), STAT2 (Pearson *R* = 0.413), STAT3 (Pearson *R* = 0.373), STAT4 (Pearson *R* = 0.277), STAT5A (Pearson *R* = 0.242), STAT5B (Pearson *R* = 0.355), and STAT6 (Pearson *R* = 0.449). We also examined the OS of STAT family members in the TCGA-KIRC database (Fig. 6B to H). Combining these findings, STAT2 emerged as the most promising candidate. We further explored the PFI and DSS associated with STAT2, finding that up-regulation of STAT2 is linked to worse survival outcomes (Fig. 6I and J). TCGA analysis revealed that STAT2 was up-regulated in tumor groups (Fig. S6A and B). Additionally, the receiver operating characteristic (ROC) curve of STAT2 demonstrated relatively high accuracy in ccRCC diagnosis (Fig. 6K). Moreover, we assessed the protein expression of STAT2 in both 786-O and 786-O-PR cells and found that STAT2 was up-regulated in 786-O-PR cells (Fig. 6L). Subsequently, we down-regulated STAT2 expression in 786-O-PR cells, resulting in relatively reductions in both mRNA and protein levels of STAT2 and SLC27A3 (Fig. 6M and N).

Based on the aforementioned results, we further investigated downstream biological behaviors. In the presence of pazopanib, after transfecting the STAT2-OE plasmid into 786-O cells, there was an enhancement in cell colony formation ability and cell viability, which could be rescued by transfection with the SLC27A3-sh plasmid (Fig. 6O and P). Similar results were observed in 769-P cells (Fig. S6C and D). Furthermore, overexpression of STAT2 in parental cells led to increased detection of LDs using BODIPY and Oil Red O staining, while subsequent knockdown of SLC27A3 inhibited LD formation (Fig. 6Q and Fig. S6E). In parental cells with STAT2 overexpression, the PINK1/Parkin mitophagy pathway was activated, which could be rescued when transfected with SLC27A3-sh plasmids (Fig. 6R and Fig. S6F). Finally, we identified 3 transcription factor binding sites for STAT2 on the SLC27A3 gene promoter from chromatin immunoprecipitation-sequencing (ChIP-seq) data in the JASPAR database: SBE1, SBE2, and SBE3 (Fig. 6S) [36]. We initially confirmed the binding of the promoter region to the STAT2 complex using a dual-luciferase reporter assay. Subsequently, we coexpressed SBE1, SBE2, and SBE3 individually with STAT2-OE plasmids and found that the fluorescence intensity was highest in ccRCC cells overexpressing SBE1. Moreover, after mutating SBE1, the fluorescence intensity decreased quite a lot(Fig. 6T). In conclusion, the transcription factor *STAT2* can up-regulate the expression of *SLC27A3* and activate downstream biological behaviors.

## Conclusion

**Fig. 7.**
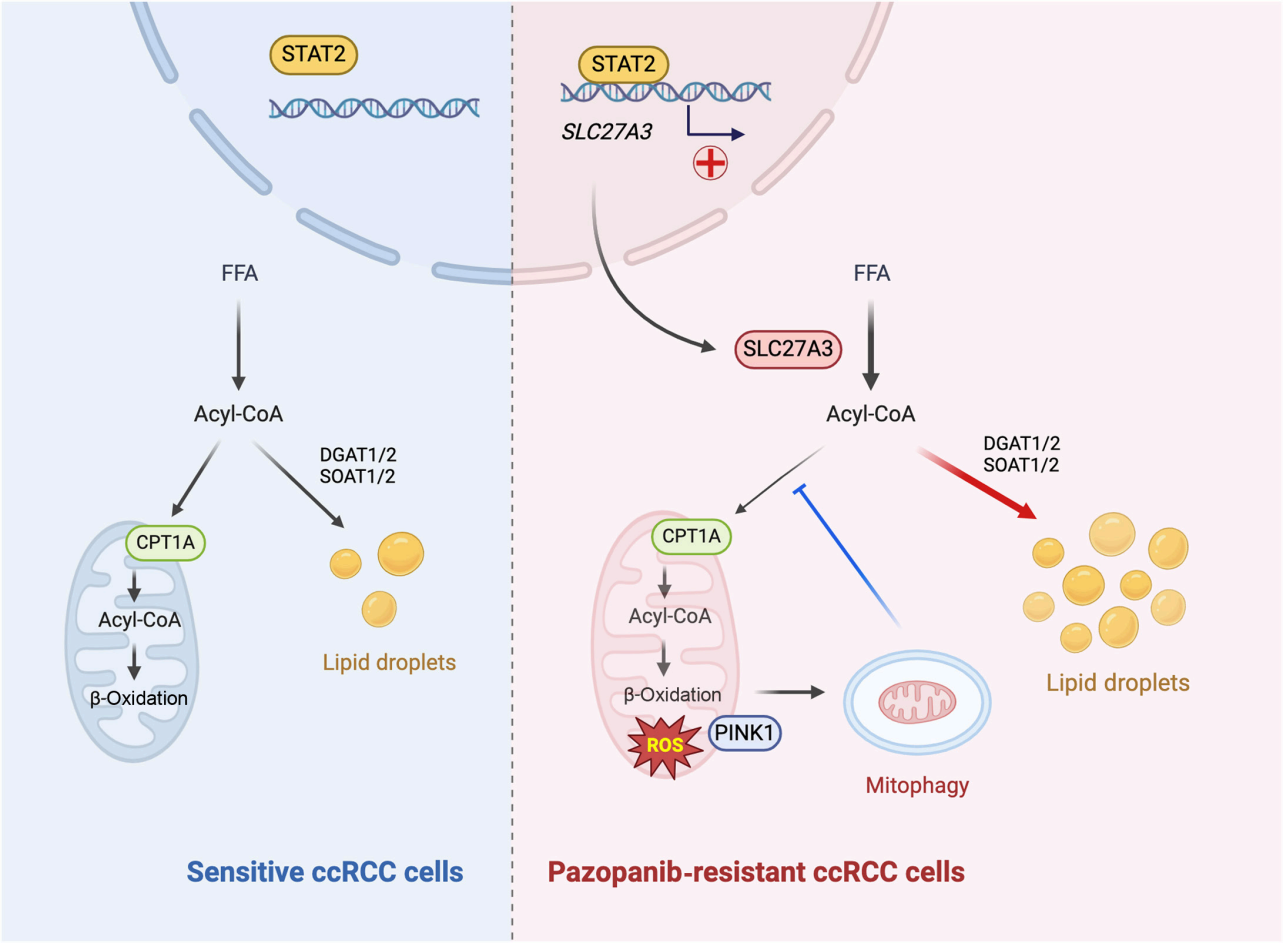
Schematic diagram.

In this study, we provided preliminary evidence that SLC27A3 is up-regulated in pazopanib resistance ccRCC and that the SLC27A3/ROS/PINK1-mediated mitophagy pathway axis plays a crucial role in pazopanib resistance. High expression of SLC27A3 produces excessive metabolites of various long-chain fatty acyl-CoA (12:0-, 16:0-, 17:0-, 20:3-CoA) to enter mitochondria for β-oxidation and produce amounts of ROS activating mitophagy. Subsequent mitophagy/ROS negative feedback controls ROS homeostasis and consumes CPT1A protein within mitochondria to suppress fatty acid β-oxidation, forcing acyl-CoA to participate in LD biosynthesis, mediating pazopanib resistance. Furthermore, STAT2 was identified as a core component of a potential upstream transcriptional factor complex for SLC27A3 (Fig. 7). Our findings shed new light on the underlying mechanism of *SLC27A3* in ccRCC TKI resistance, which may provide a novel therapeutic target for the management of ccRCC.

## Discussion

SLC27A3 is an acyl-CoA ligase that catalyzes the conversion of LCFAs and very long-chain fatty acids (VLCFAs) into fatty acyl-CoA [24]. Acyl-CoA is crucial in various metabolic processes, including β-oxidation, lipid synthesis, fatty acid modification, and posttranslational modification [26]. Tumor cells often reprogram these metabolic activities to meet their tumorigenic needs or in response to external pressures [37]. SLC27A3, originally termed very long-chain acyl-CoA synthetases (ACSVL3), was reported to mediate the activation of C16:0 and C24:0 fatty acids in mice without any detected fatty acid transport function [28]. It has been linked with tumorigenesis of malignant cancers [24,29,30,38] and autism [27], and is involved in onco-sphingolipid metabolism, although further research is needed. Herein, we provide hitherto undocumented evidence of SLC27A3 in ccRCC and its association with TKI resistance. Lipidomic analysis of ccRCC cell lines identified C12:0-, C16:0-, C17:0-, and C20:3-LCFAs as potential substrates of SLC27A3, consistent with existing research. This suggests that the corresponding acyl-CoAs contribute to ccRCC TKI resistance. Moreover, as shown in Fig. 1K, SLC27A3 is mainly highly expressed in lipid-enriched tumors such as ccRCC and glioblastoma, while the expression of SLC27A3 did not increase or even decrease in other tumors such as breast cancer and lung cancer, indicating that SLC27A3 is certain lipid tumor specific, which is the main feature that distinguishes it from other oncogenes in ccRCC. The tumor specificity of SLC27A3 in ccRCC is an important prerequisite for the development of targeted drugs.

Acyl-CoA can be transported into mitochondria via CPT1A for adenosine 5′-triphosphate (ATP) production or utilized for LD biosynthesis independently of mitochondria. It has been reported that CPT1A pumps fatty acids into mitochondria to consume LDs in ccRCC, leading to tumor growth restriction [12]. This suggests that LD synthesis after fatty acid activation in ccRCC is more conducive to tumor survival than β-oxidation of fatty acids for energy production. LDs can provide sufficient raw materials for biofilm synthesis, promoting tumor proliferation, and can effectively prevent cell damage from lipotoxicity due to lipid peroxidation by sequestering unsaturated fatty acids [23]. In the presence of drugs, tumor cells may reduce metabolic efficiency in response to external pressure [39]. This adaptation serves 2 purposes: ensuring adequate raw materials for proliferation and division while avoiding excessive by-products that can cause cellular damage. The metabolic reprogramming of fatty acyl-CoA in drug-resistant cells might be a promising avenue for overcoming clinical drug resistance challenges, which is essential to be clarified in future study.

Mitochondria are the primary site of ROS production, occurring alongside β-oxidation and oxidative phosphorylation (OXPHOS) [40]. Tumor cells are characterized by excessive ROS production, which can drive gene expression and pathway regulation [41]. ROS is a double-edged sword, and its dynamic homeostasis is crucial for promoting the occurrence and progression of cancers. Appropriate ROS levels can enhance genomic instability in tumor cells and activate tumor-promoting signaling pathways, while excessive ROS can increase the oxidative stress load, eventually leading to cell death [42][38]. In our study, we found that overexpression of SLC27A3 in pazopanib-resistant cells resulted in ROS accumulation without cell death. Specifically, SLC27A3 overexpression can produce increased amounts of fatty acyl-CoA for ATP production and lipid synthesis. Due to the stability of mitochondrial numbers in cells, these oxidative phosphorylated substrates increase the mitochondrial load, accompanied by the production of by-products like ROS, which can decline MMP and affect mitochondrial function. A certain level of ROS can be buffered by the glutathione (GSH) system [43], but when the threshold is exceeded, a vicious cycle can arise, eventually leading to apoptosis. Pazopanib-resistant cells may activate new regulatory mechanisms to adapt to this change, as suggested by KEGG and GO analysis from RNA sequencing in our research, indicating that mitophagy might be one such mechanism.

Cellular mitophagy can selectively degrade damaged or dysfunctional mitochondria to maintain mitochondrial homeostasis [44]. It is also linked to tumor drug resistance [21,22]. In this study, we demonstrated that SLC27A3 overexpression could activate the PINK1/Parkin mitophagy pathway. ROS produced from acyl-CoA catalyzed by SLC27A3 in mitochondria damages MMP, preventing PINK1 from degradation and thus allowing its stable accumulation on the mitochondrial outer membrane to initiate mitophagy [45]. Mitophagy, in synergy with SLC27A3, participated in the process of pazopanib resistance. As demonstrated in this research, mitophagy can control ROS levels to prevent ROS-induced apoptosis while maintaining its function of activating tumor-promoting signaling. Additionally, mitophagy itself can provide substrates for cell survival and proliferation, particularly under pazopanib-induced pressure. Furthermore, mitophagy can effectively suppress β-oxidation and OXPHOS by reducing mitochondrial mass and CPT1A protein levels, forcing acyl-CoA into the process of LD storage. We also found that LDs mediated TKI resistance in ccRCC. Due to the lack of specific inhibitors against LD synthesis, we combined DGAT1/2 and SOAT1/2 as an LD synthesis inhibitor cocktail (LDIC), considering that TG and CE are the main components of LDs [9,10]. Mitophagy might be a potential target for overcoming TKI resistance of ccRCC. While there is a lack of specific mitophagy inhibitor, autophagy inhibitor like 3-MA and chloroquine are now substitutes to it. The combination of mitophagy/autophagy inhibitor and sunitinib/pazopanib is expected to be tested in the preclinical and clinical trial for TKI resistance therapy in ccRCC.

STAT2 belongs to the signal transducer and activator of transcription family, which typically forms a heterodimer transcriptional complex with STAT1 [33]. Our study revealed no correlation between STAT1 and SLC27A3 expression or the clinical prognosis of ccRCC, while STAT2 demonstrated meaningful results. Although some studies have shown STAT1’s involvement in immunology evasion of RCC [46], it may play a synergistic role with STAT2 in the process of pazopanib resistance in ccRCC. Given the current use of TKI and immunotherapy as first-line treatments for advanced ccRCC [47], the roles of STAT2, STAT1, and their transcriptional factor complex in ccRCC drug resistance warrant further investigation and will be a potential therapeutic target, even including the upstream of STAT family, Janus kinase (JAK).

## Materials and Methods

### Patient specimens and cell lines

Ethical approval was obtained from the Institutional Review Board of the First Affiliated Hospital of Zhejiang University School of Medicine prior to sample collection. ccRCC and adjacent normal tissue specimens were acquired from patients who underwent radical nephrectomy for RCC diagnosis at the Department of Urology of the First Affiliated Hospital of Zhejiang University School of Medicine between 2021 and 2024. HK-2, HEK-293T, 769-P, Caki-1, 786-O, and A498 cell lines were obtained from the Chinese Academy of Sciences Shanghai Cell Bank (Shanghai, China). 786-O, 769-P, and Caki-1 cells were cultured in RPMI 1640 medium (Hyclone, USA), HK-2 and A498 cells were cultured in RPMI minimum essential medium (MEM) (Hyclone, USA), and HEK-293T cells were cultured in Dulbecco’s modified Eagle’s medium (DMEM) (Procell, Wuhan, China) supplemented with 10% fetal bovine serum (Biologic Industries, Israel) and 1% penicillin/streptomycin (Servicebio, Wuhan, China). All cell lines were maintained in a humidified atmosphere at 37 °C with 5% CO_2_.

### Detection of cell cycle

Cells were fixed overnight in ethanol at −20 °C, followed by centrifugation at 1,500 rpm for 6 min to harvest the cells. The cells were then stained with propidium iodide (PI). The percentage of cells in each phase (G_0_-G_1_, S, and G_2_-M) was evaluated using flow cytometry (BD FACSCalibur, USA).

### Colony formation

786-O-PR, 786-O, and 769-P cells were seeded into 6-well plates at a density of 700 cells per well, with a drug-containing medium added for culture. Plates were incubated at 37 °C in an atmosphere of 5% CO_2_ for 12 d. Cells were fixed with 4% paraformaldehyde and stained with 0.1% crystal violet. Colonies consisting of more than 50 cells were considered positive[48].

### Pazopanib-resistant cell line 786-O-PR establishment

Parental 786-O cells were stably passaged at least 5 times after being exposed to pazopanib hydrochloride (MedChemExpress, USA) at an initial concentration of 2 μM. The pazopanib hydrochloride concentration was gradually increased over 6 months to establish the stable pazopanib-resistant cell line 786-O-PR. The concentration gradient used during this process was as follows: 2, 4, 8, 10, and 12 μM.

### Quantitative real-time PCR

Total RNA was extracted using RNAiso Plus Reagent (Takara) and converted to cDNA. Quantitative real-time polymerase chain reaction (PCR) was conducted with SYBR qPCR Mix (Vazyme, China) on a Bio-Rad CFX96 Detection System, following standard protocols. qPCR primer pairs were synthesized by Tsingke Bio (Beijing, China), and their sequences are listed in Table S1. β-Actin served as the internal control.

### Western blot

Cells were lysed using radioimmunoprecipitation assay buffer (Fude Bio, Hangzhou, China) for total protein extraction. Following denaturation, proteins were separated by sodium dodecyl sulfate–polyacrylamide gel electrophoresis in the running buffer and transferred to polyvinylidene difluoride (PVDF) membranes (Millipore, USA) in the transfer buffer. A 5% nonfat milk solution in tris-buffered saline containing Tween 20 was used to block the PVDF membrane for 2 h. After washing away excess milk, the membrane was incubated with corresponding primary antibodies overnight at 4 °C. The following day, a secondary antibody conjugated to horseradish peroxidase (1:4,000, catalog nos.: GB23301 and GB23303; Servicebio, Wuhan, China) was used to bind the primary antibody. A list of primary antibodies is provided in Table S4.

### RNA sequencing

Full transcriptome sequencing and bioinformatics analyses were conducted using UMI RNA-seq technology (Seqhealth, Wuhan, China).

### Immunohistochemistry

Clinical samples from patients with RCC and corresponding adjacent normal tissues were fixed for immunohistochemistry, as previously described [39]. Additionally, orthotopic transplantation models were established in vivo and fixed for immunohistochemical analysis. Clinical samples were stained using a SLC27A3 primary antibody, while orthotopic tumor tissues were stained with both SLC27A3 and Ki-67 primary antibodies. H-score was used for quantitation.

### Cell viability assay

Five thousand cells were seeded into each well of 96-well plates and cultured in a complete medium. After 24 h, 110 μl of CCK-8 reagent (Fude Bio, Hangzhou, China) was added to each well, and the plates were incubated for 1 h at 37 °C. Cell viability was assessed for 4 d using a microplate reader (ELx800, Bio-Tek, USA) to measure absorbance at 450 nm[49].

### Small interfering RNA, plasmid transfection, and lentiviral constructs

Small interfering RNAs (siRNAs) for knockdown were obtained from Tsingke Bio (Beijing, China). Plasmids for knockdown or overexpression were acquired from Vigene Biosciences (Shandong, China), and dual-luciferase assay reagents were purchased from Ruipute Biotechnology (Hangzhou, China). Following protocols, siRNA, plasmids, and vectors were transfected into 786-O-PR, 786-O, and 769-P cells using Lipofectamine 3000 or JetPRIME transfection agents (Polyplus, USA). 786-O-PR, 786-O, and 769-P cells were also infected with lentivirus targeting SLC27A3 knockdown. Lentivirus production was achieved by transfecting 293T cells with psPAX2, pMD2G, and target plasmids.

### Oil Red O staining

Cells were fixed with 4% paraformaldehyde, and LDs were visualized using a Modified Oil Red O Staining Kit (Beyotime Institute of Biotechnology, China) following the manufacturer’s protocol.

### BODIPY staining

Cells were fixed using 4% polyformaldehyde. LDs were detected with the BODIPY 493/503 probe (D3922, Thermo Fisher Scientific, USA) following the manufacturer’s protocol. Nuclei were stained with 4′,6-diamidino-2-phenylindole (Servicebio, Wuhan, China). Data analysis was performed using the STELLARIS 5 Scanning Confocal Microscope and its associated software (Leica, France).

### Detection of MMP

Cells were seeded in a glass-bottom cell culture dish (BS-15-GJM, biosharp, China). The JC-1 probe was employed to assess the MMP of living cells using the JC-1 MitoMP assay (MT09, DOJINDO, Japan). Subsequently, data analysis was performed using a STELLARIS 5 Scanning Confocal Microscope.

### Detection of ROS

2′,7′-Dichlorodihydrofluorescein diacetate (DCFH-DA) fluorescent probes (S0033M, Beyotime Institute of Biotechnology), diluted in serum-free medium, were incubated with cells at 37 °C for 30 min. Subsequently, the cells were harvested and transferred to a flow tube. Fluorescence was collected and analyzed using a flow cytometer.

### Transmission electron microscope

Cells were fixed using an electron microscope fixative (G1102, Servicebio, China) following established protocols. TEM was employed to visualize mitophagy as previously described [50].

### Mitophagy probe detection

Cells were seeded in a glass-bottom cell culture dish (BS-15-GJM, biosharp, China), and the mitophagy phenomenon was assessed using Mtphagy Dye (MD01, DOJINDO, Japan) following the manufacturer’s protocol.

### Luciferase reporter assays

Renilla reporter plasmids and pGL3-basic containing the SLC27A3 promoter were cotransfected with Vector or STAT2 overexpression plasmids in 786-O and 769-P ccRCC cells. The fluorescence intensity ratio was used to assess the binding affinity of the STAT2 complex to the SLC27A3 promoter. Potential transcriptional binding sites were identified using the JASPAR database. Dual-Luciferase Reporter Assay System (Promega, USA) was employed to measure luciferase activities according to the manufacturer’s specifications [39].

### Orthotopic transplantation tumor model

Pazopanib hydrochloride was used for tumor treatment in stable 786-O-PR cell lines infected with lentivirus containing shSLC27A3/Vector and transfected with the luciferase reporter gene. These cells were established for renal subcapsular orthotopic tumor formation. d-Luciferin (150 mg/kg) diluted in phosphate-buffered saline was injected intraperitoneally for tumor monitoring using In-Vivo FX PRO, following a previously described protocol [51]. All animal experiments were conducted in accordance with the institutional guidelines approved by the First Affiliated Hospital, Zhejiang University School of Medicine.

### Bio-informatics research

Data from the GEO database (GDS505), the KIRC group from the TCGA database, THPA database, and the Xiantao online analysis database were utilized in this study.

### LD synthesis inhibitor

An LD synthesis inhibitor cocktail was prepared by combining Avasimibe, A-922500, and PF-06424439 (obtained from MedChemExpress, USA) at their respective median inhibitory concentration (IC_50_) concentrations.

### Micro-targeted lipidomic of acyl-CoAs

LipidALL Co. Ltd. provided technical support and data analysis for this study. LipidALL Technologies conducted an analysis of acyl-CoAs, as previously described [52], to identify potential differentially metabolized substrates of SLC27A3.

### Nontargeted lipid metabolomics

This research was supported by Lianchuan Biomedical Technology Co. Ltd. to identify potential differential metabolites following the regulation of SLC27A3 expression.

### Statistical analysis

All in vitro experiments were replicated at least 3 times. Each of the in vivo groups contains 5 BALB/c-nu mice. Survival outcomes were evaluated by Kaplan–Meier analysis and compared using the log-rank test. Statistical analyses were performed using Prism 10.0 (GraphPad) and SPSS 22.0 (IBM Corporation) software. All data were presented as mean ± standard deviation (SD). Unpaired parameter Student’s *t* tests and nonparametric tests were used to calculate *P* values. Specifically, we first tested for normality using the normal test function in Prism10.0 and SPSS 22.0 to ensure that parameter *t* test is applicable. Nonparametric tests were used for data that did not meet the normal distribution. Then, Welch’s correction was used to adjust data if the statistical significance of *F* test in parameter *t* test was at a *P* value of less than 0.05. Differences were considered statistically significant at a *P* value of less than 0.05. No statistical significance was presented as ns (*P < 0.05, **P < 0.01, ***P < 0.001).

## Data Availability

The data that support the findings of this study are available in the Supplementary Materials.
