# Supplementary material for: STAT2/SLC27A3/PINK1-Mediated Mitophagy Remodeling Lipid Metabolism Contributes to Pazopanib Resistance in Clear Cell Renal Cell Carcinoma
Source: Research (Wash D C). 2024 Nov 26;7:0539. doi: 10.34133/research.0539 (PMC11588985; doi:10.34133/research.0539)
Supplement: Supplementary 1 — Figs. S1 to S6 Tables S1 to S5 [file research.0539.f1.zip › Supplemental Information.docx]

**Supplementary Table 1: qRT-PCR primer sequences in this study**

| Gene names | Primer sequence |
| --- | --- |
| β-Actin-F | 5′- CATGTACGTTGCTATCCAGGC-3′ |
| β-Actin-R | 5′- CTCCTTAATGTCACGCACGAT-3′ |
| SLC27A3-F | 5′- CCCTGCTGGAATTAGCGATTT -3′ |
| SLC27A3-R | 5′- GGGCGAGGTAGATCACATCTT -3′ |
| STAT2-F | 5′- GAGCCAGCAACATGAGATTGA -3′ |
| STAT2-R | 5′- GCCTGGATCTTATATCGGAAGCA -3′ |

**Supplementary Table 2: siRNA/shRNA sequences in this study**

| Name | Sequence |
| --- | --- |
| SLC27A3-sh1 | 5′- GGAAGATGTGATCTACCTCGCTCAAGAGGCGAGGTAGATCACATCTTCC-3′ |
| SLC27A3-sh2 | 5′- TGTGCCGATACCTTGTCAACCTCAAGAGGGTTGACAAGGTATCGGCACA-3′ |
| STAT2-si1 | 5′- GCTGAGCCATAGGTCTAAATA-3′ |
| STAT2-si2 | 5'-TGTCTTCTGCTTCCGATATAA-3′ |
| PINK1-sh1 | 5′-CGGCTGGAGGAGTATCTGATATCAAGAGTATCAGATACTCCTCCAGCCG-3′ |
| CPT1A-sh | 5′-GCCATGAAGCTCTTAGACAAATCAAGAGTTTGTCTAAGAGCTTCATGGC-3′ |

**Supplementary Table 3: Drugs and reagents**

| Drug / Reagent | Source | Identifier / formulation |
| --- | --- | --- |
| Pazopanib Hydrochloride | MedChemExpress | Catalog No: HY-12009 |
| A 922500 | MedChemExpress | Catalog No: HY-10038 |
| PF-06424439 methanesulfonate | MedChemExpress | Catalog No: HY-108341A |
| Avasimibe | MedChemExpress | Catalog No: HY-13215 |
| BODIPY™ 493/503 | ThermoFisher | Catalog No: D3922 |
| Lipofectamine™ 3000 | ThermoFisher | Catalog No: L3000075 |
| jetPRIME ® | Polyplus-transfection ® | Catalog No:101000001 |
| Mtphagy Dye | DOJINDO | Catalog No:MT02 |
| Modified Oil Red O Staining Kit | Beyotime Biotechnology | Catalog No:C0158M |

**Supplementary Table 4： Primary and secondary antibodies used in this study**

| Antibody | Source | Identifier | Host |
| --- | --- | --- | --- |
| SLC27A3 | Proteintech | Catalog No: 12943-1-AP | Rabbit |
| Parkin | Abcam | Catalog No: ab77924 | Mouse |
| PINK1 | Abcam | Catalog No: ab216144 | Rabbit |
| PINK1 | Proteintech | Catalog No: 23274-1-AP | Rabbit |
| LC3B | Proteintech | Catalog No: 14600-1-AP | Rabbit |
| CPT1A | CST | Catalog No: 12252 | Rabbit |
| TOM20 | Proteintech | Catalog No: 66777-1- Ig | Mouse |
| COXIV | Proteintech | Catalog No:11242-1-AP | Rabbit |
| STAT2 | CST | Catalog No:72604 | Rabbit |
| STAT2 | Proteintech | Catalog No:16674-1-AP | Rabbit |
| Ki-67 | ABclonal | Catalog No: A23722 | Rabbit |
| β-actin | Proteintech | Catalog No: 66009-1-Ig | Mouse |
| HRP conjugated Goat Anti-Mouse IgG (H+L) | Servicebio | Catalog No: GB23301 | Goat |
| HRP conjugated Goat Anti-Rabbit IgG (H+L) | Servicebio | Catalog No: GB23303 | Goat |

**Supplementary Table 5：Mutant plasmid sequences used in double luciferase reporter gene experiments**

| Name | Sequence |
| --- | --- |
| SBE1-mut | TTAGGAGGGGGAA |
