# Supplementary figures and images for: STAT2/SLC27A3/PINK1-Mediated Mitophagy Remodeling Lipid Metabolism Contributes to Pazopanib Resistance in Clear Cell Renal Cell Carcinoma
Source: Research (Wash D C). 2024 Nov 26;7:0539. doi: 10.34133/research.0539 (PMC11588985; doi:10.34133/research.0539)

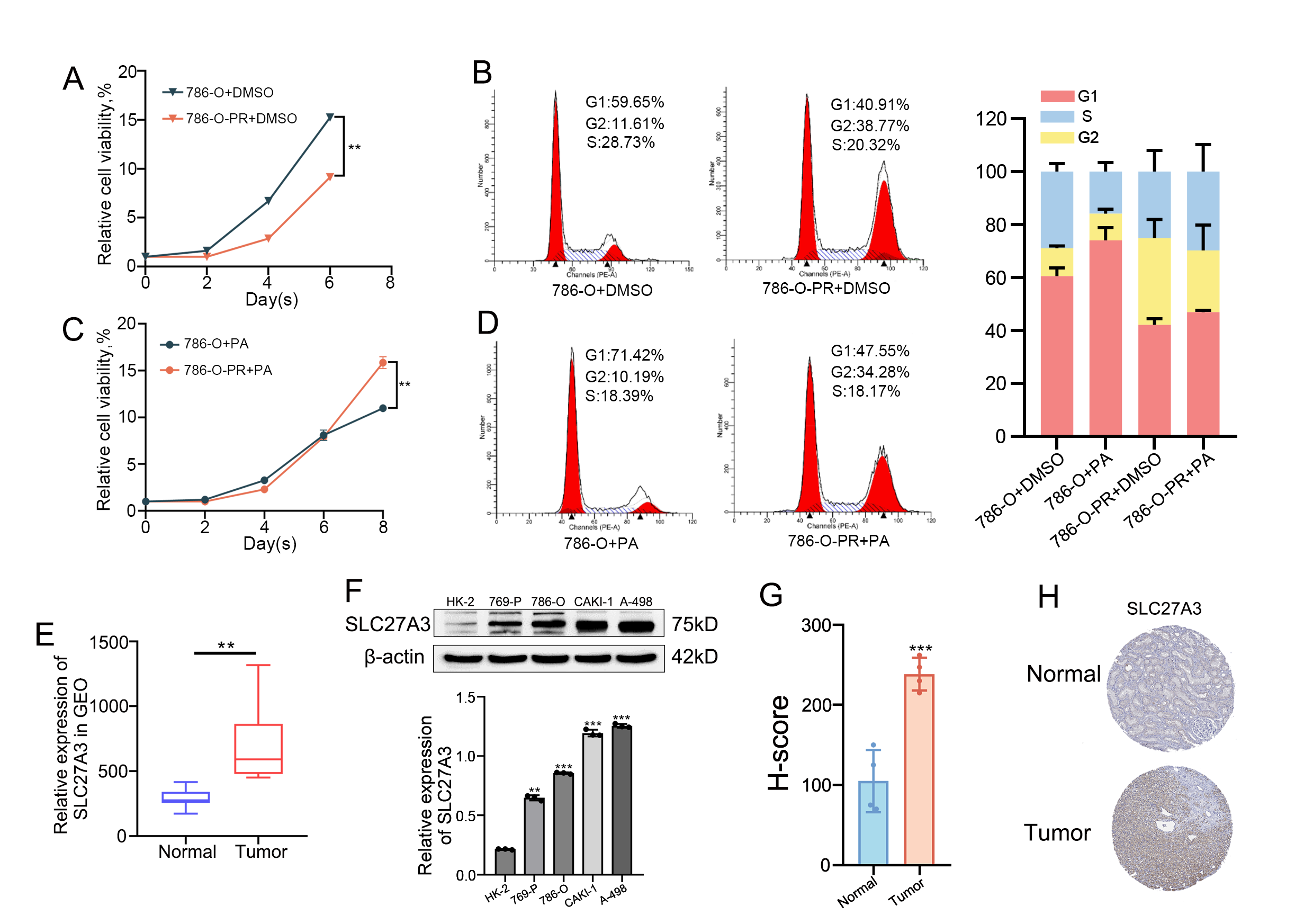

Supplement: Supplementary 1 — Figs. S1 to S6 Tables S1 to S5 [file research.0539.f1.zip › FigureS1.tif]

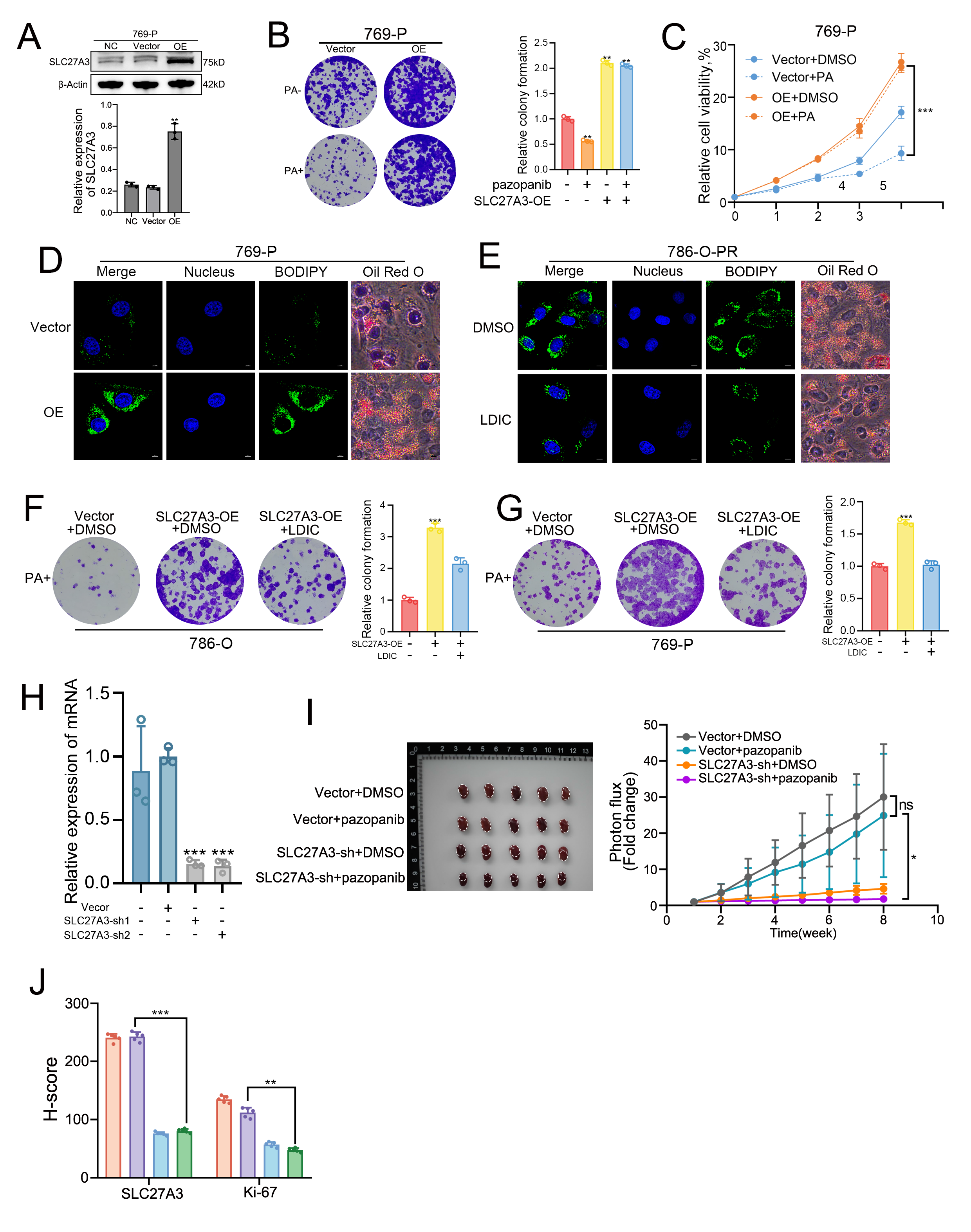

Supplement: Supplementary 1 — Figs. S1 to S6 Tables S1 to S5 [file research.0539.f1.zip › FigureS2.tif]

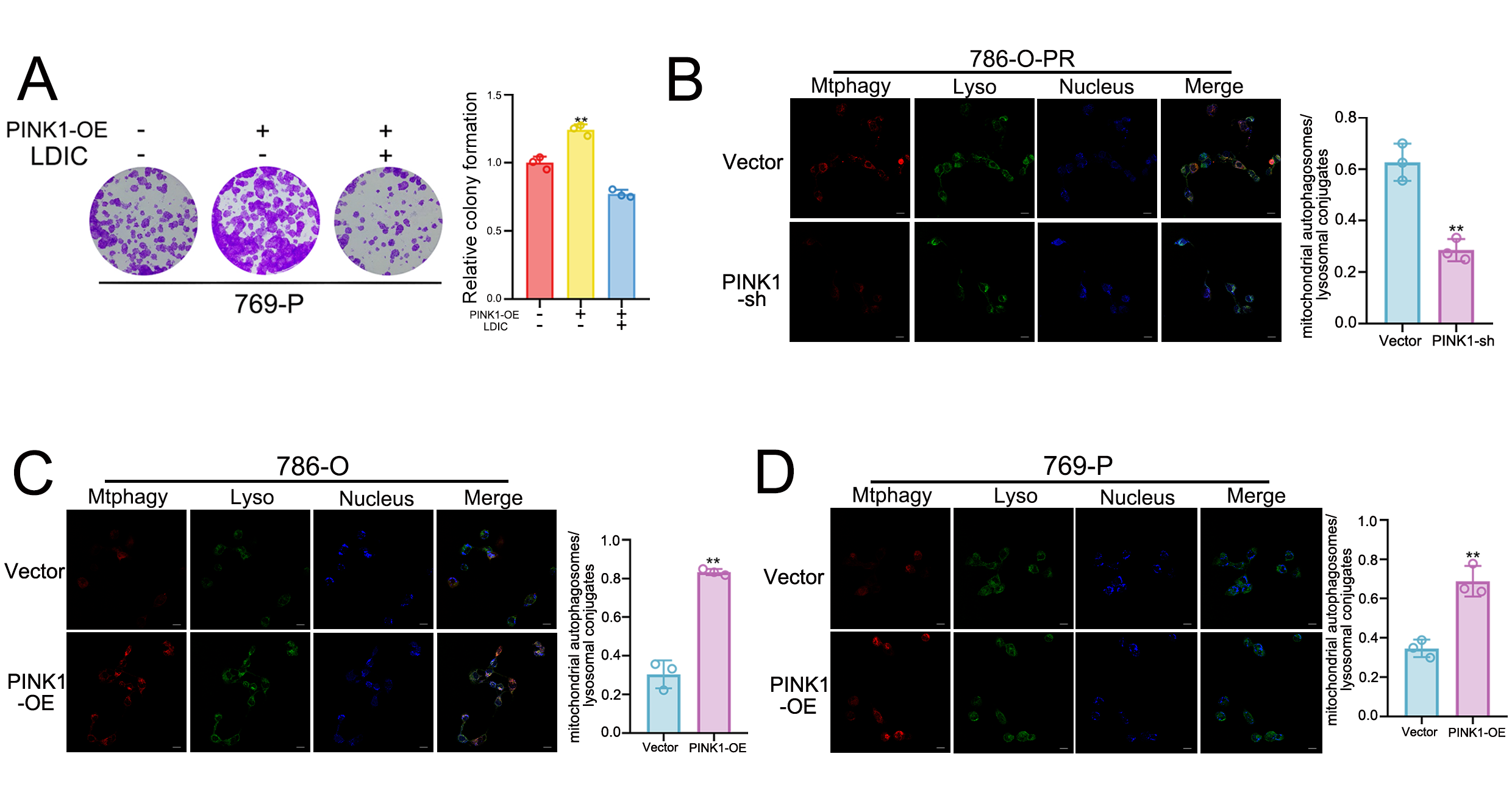

Supplement: Supplementary 1 — Figs. S1 to S6 Tables S1 to S5 [file research.0539.f1.zip › FigureS3.tif]

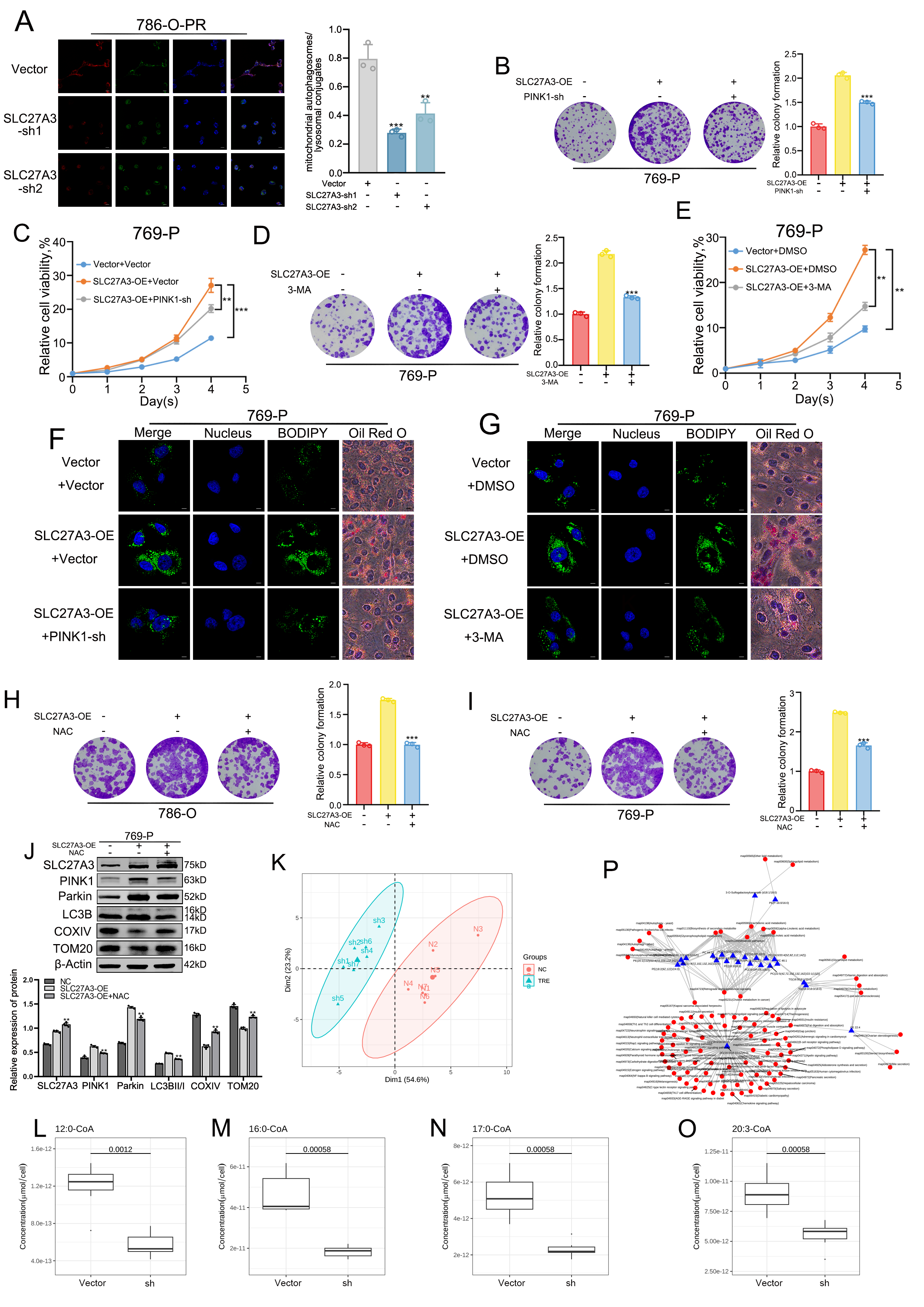

Supplement: Supplementary 1 — Figs. S1 to S6 Tables S1 to S5 [file research.0539.f1.zip › FigureS4.tif]

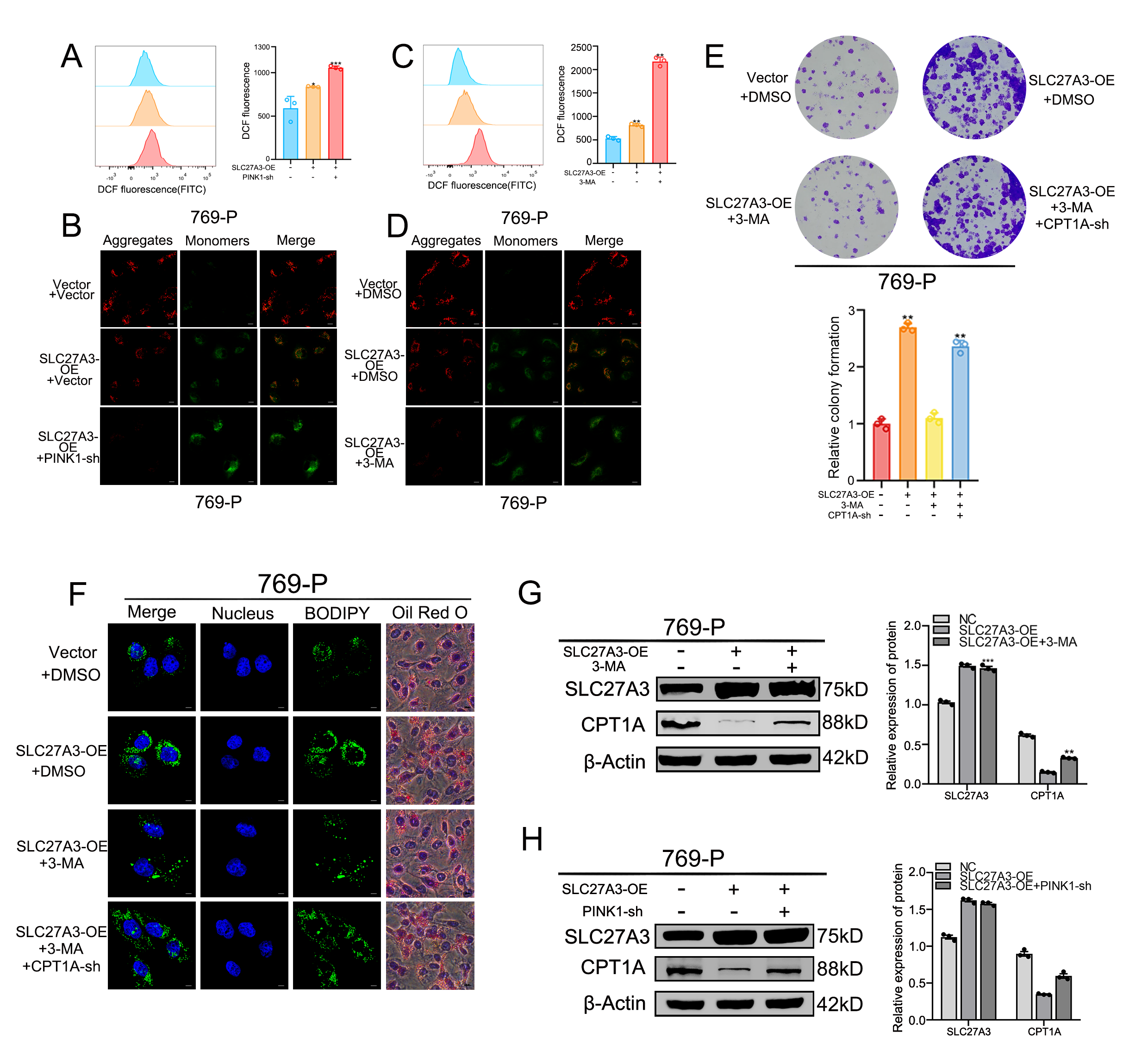

Supplement: Supplementary 1 — Figs. S1 to S6 Tables S1 to S5 [file research.0539.f1.zip › FigureS5.tif]

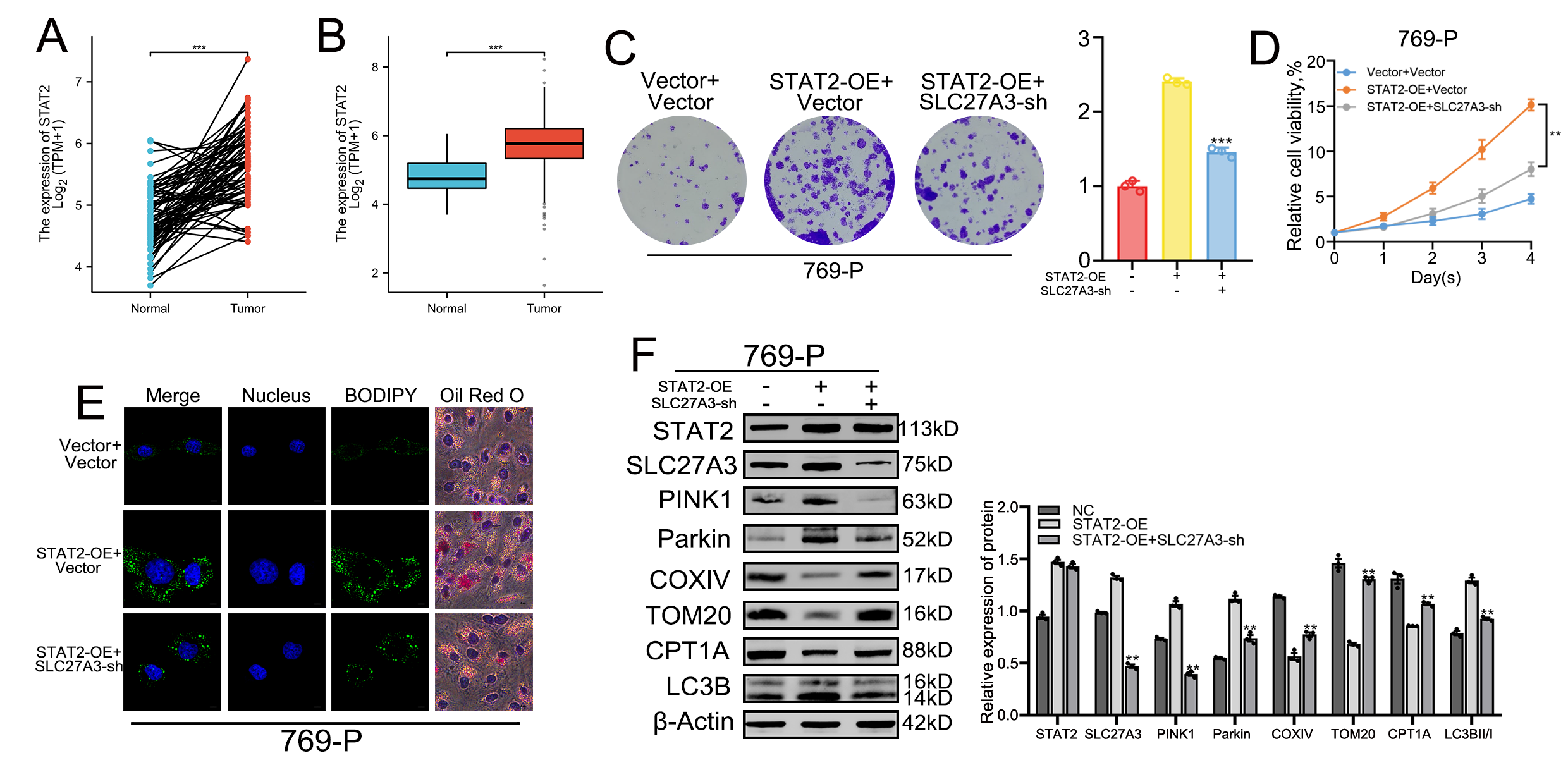

Supplement: Supplementary 1 — Figs. S1 to S6 Tables S1 to S5 [file research.0539.f1.zip › FigureS6.tif]
